# Poly(glycerol sebacate): A Comparative Study of Various Synthesis Methods

**DOI:** 10.1021/acs.biomac.5c01548

**Published:** 2025-10-04

**Authors:** Silke Andrä-Żmuda, Paweł Chaber, Magdalena Martinka Maksymiak, Marta Musioł, Grażyna Adamus

**Affiliations:** 111480 Centre of Polymer and Carbon Materials, Polish Academy of Sciences, 34, M. Curie-Skłodowska Street, 41-819 Zabrze, Poland

## Abstract

This paper compares five synthesis methods for poly­(glycerol sebacate) (PGS) prepolymers: high-temperature polycondensation, classical polycondensation under reduced pressure, enzymatic synthesis using *Candida antarctica* lipase B (CALB), enzymatic synthesis in the presence of acetone as a solvent, and Amberlyst-15-catalyzed polycondensation. All reactions were performed in the same laboratory to eliminate variability resulting from differences in instrumentation and experimental conditions. The obtained PGS samples were analyzed using FTIR, NMR, ESI-MS, GPC, DSC, and TGA. The enzymatic synthesis with CALB provided the best control of the reaction process, prevented gelation, and produced prepolymers with higher molecular weights and narrow dispersity. Structural analyses by NMR and ESI-MS revealed the presence of both linear and branched PGS structures. The obtained results clearly confirm that the synthesis strategy significantly influences the molecular architecture and physicochemical properties of the resulting PGS prepolymer. These findings provide a basis for further design of PGS-based materials for biomedical application.

## Graphical abstract

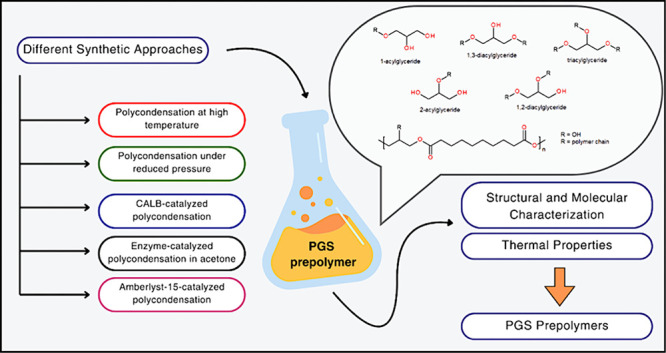

## 1 Introduction

One of the main challenges of modern medical chemistry is the constant search for new materials for medical applications which are characterized by high biocompatibility to the human body, good biodegradability, and nontoxicity. An interesting candidate fulfilling the above-mentioned requirements could be poly­(glycerol sebacate) (PGS). This polyester is usually obtained through the polycondensation reaction of glycerol and sebacic acid. The obtained poly­(glycerol sebacate) exhibits predominantly hydrophobic character due to the long aliphatic chains of sebacic acid. However, the presence of hydroxyl groups from the glycerol units in the polymer backbone contributes to the limited hydrophilic character of PGS.^1^ What is worth mentioning is that glycerol is the main building block of lipids, while sebacic acid is a natural intermediate metabolic compound that appears during the ω-oxidation of fatty acids in the human body.^2^ Furthermore, glycerol-based polymers and some polymers containing sebacic acid are accepted by the Food and Drug Administration (FDA), which makes PGS a promising candidate for medical applications.^3^ Due to its flexible and elastomeric nature, poly­(glycerol sebacate) (PGS) has also been reported to exhibit shape memory properties.^4^ This makes it suitable for soft tissue engineering applications and has initiated research into the potential use of PGS in areas such as skin, nerve, cardiac, and cartilage tissue engineering.^5–8^ Another advantage is the possibility to tailor the mechanical properties and degradation rates to make the PGS material suitable for a specific destined application.^1^ Since the synthesis of PGS was first mentioned in 2002 by Wang et al.,^9^ many studies have indicated a wide range of possibilities for its use, not only in tissue engineering applications but also in drug delivery and wound healing.^10,11^

PGS has been intensively studied for over 20 years; therefore, various methods have been used to obtain this polymer. However, three main approaches can be distinguished: (1) traditional polycondensation under reduced pressure, (2) enzyme-catalyzed polycondensation, and (3) microwave assisted synthesis.^12^ The PGS synthesis offers several advantages, making this polyester a particularly suitable and attractive material for medical applications. First, the polycondensation does not require the use of solvents, which eliminates the need to separate the obtained PGS prepolymer from the reaction medium. Second, no hazardous catalysts are necessary during the reaction. However, the traditional polycondensation of glycerol with sebacic acid is typically carried out under relatively harsh conditions, involving high temperatures (above 120 °C) and a vacuum. Moreover, the reaction is time-consuming, often requiring several hours, and only oligomers are formed during the prepolymerization step to prevent gelation. Therefore, there have been several attempts to modify the traditional PGS synthesis pathway to overcome these limitations.

The most common and well-described reaction in the literature is the traditional polycondensation of glycerol with sebacic acid under reduced pressure. This synthesis route is usually divided into two steps. The first is the prepolymerization stage, during which a mostly linear oligomer, known as the PGS prepolymer, is obtained. The second step is cross-linking, where a flexible three-dimensional cross-linked PGS polymer is formed. This cross-linking is usually carried out at high temperatures under vacuum conditions.^13^ Additionally, some researchers report that the use of a chemical agent or radiation could promote the cross-linking of the prepolymerized PGS.^14^ However, one potential drawback of the highly cross-linked PGS polymer system is the resulting resistance to dissolution, which makes it difficult to further modify the polymer structure, for example, by grafting other functional fragments and molecules into the network. The second method discussed for PGS synthesis involves an enzyme-catalyzed reaction in which immobilized *Candida antarctica* lipase B (CALB) is used to obtain PGS polyesters.^15–17^ An enzyme-catalyzed reaction offers several advantages over the traditional reactions including (1) high catalyst selectivity, (2) the possibility of reusing the CALB catalyst for following reactions, (3) milder reaction conditions compared to conventional polycondensation under reduced pressure, and (4) reduced cross-linking, which enables the synthesis of PGS with higher molecular weight.^16,18^ The third discussed in the literature PGS synthesis method involves the polycondensation of glycerol and sebacic acid assisted by microwaves. One of the biggest advantages of this reaction route is the drastic decrease in reaction time (from several days to even 15 min). However, a significant glycerol loss is observed, which significantly lowers the control over the initial starting monomer ratio and, therefore, influences the properties of the obtained product and often makes it stiff after curing.^19^ Amberlyst-15 (A-15) is an interesting potential catalyst for the synthesis of PGS. It is a strongly acidic, macroporous ion-exchange resin based on cross-linked polystyrene and functionalized with sulfonic groups (−SO_3_H), which exhibits the properties of Brønsted acids. The heterogeneous catalyst has gained increasing attention in organic synthesis due to its outstanding properties, which include (1) relatively low cost, (2) the possibility of catalyst recovery, (3) easy separation from the reaction medium, and (4) operational simplicity. Several studies have shown the high potential of this catalyst in various reactions such as esterification, etherification, cyclization, and electrophilic aromatic substitution.^20^ To our knowledge, only Thongkam et al. have applied Amberlyst-15 for the synthesis of PGS. They compared in their study results of A-15 catalysis with the impact of zinc acetate and zinc oxide on the outcoming PGS sample.^21^

Despite extensive research on the synthesis and potential biomedical applications of PGS, there are very few publications comparing different synthetic strategies for this polyester in a single laboratory. This is an important issue, discussed, among others, by Li et al.^22^ These authors pointed out that PGS obtained under similar reaction conditions by different research groups exhibits varying properties. These differences may result from such factors as the purging flow rate of the inert gas, the capacity of the vacuum pump, or even temperature uniformity inside vacuum ovens.^23^ Therefore, in our opinion, it is worthwhile to compare the chemical properties of different PGS prepolymers obtained within a single laboratory by using various synthetic methods. This may help assess the impact of the synthesis route on the resulting PGS product and at the same time identify the synthetic method that may be optimal for obtaining a PGS prepolymer suitable for further biomedical applications.

The aim of this study was to optimize and compare different literature-reported methods for synthesizing poly­(glycerol sebacate) (PGS) prepolymer, in order to evaluate how the choice of synthesis strategy influences the properties of the final product. All syntheses were performed within a single laboratory to minimize discrepancies arising from variations in experimental conditions and equipment, as highlighted by Li et al.^22^ and others. Two of the three most commonly reported reaction pathways were selected: traditional polycondensation under reduced pressure and the enzymatic reaction catalyzed by *Candida antarctica* lipase B (CALB). Additionally, inspired by the work of Perin et al.^18^ we investigated the effect of conducting the prepolymerization in the presence of acetone as a solvent. A high-temperature synthesis, based on the procedure of Saudi et al.,^24^ was also carried out due to its relatively short reaction time. Moreover, a polycondensation reaction catalyzed by the heterogeneous Amberlyst-15 catalyst was performed. We anticipate that the comparative approach used in this work should enable the evaluation of the effectiveness of selected methods in the production of a PGS prepolymer suitable for specific biomedical applications.

## 2 Experimental Section

### 2.1 Materials

All of the reactants were used without further purification. Sebacic acid, acetone, and lipase acrylic resin from *Candida antarctica* were purchased from Sigma-Aldrich. Bidistilled glycerol was obtained from VWR, and chloroform was purchased from J.T. Baker. Molecular sieves 4 Å (POCH) and Amberlyst-15 hydrogen form (Fluka Analytical) were dried in a vacuum oven prior to use.

### 2.2 Methods

#### 2.2.1 Nuclear Magnetic Resonance (NMR)

The NMR analysis was conducted on a Bruker-Avance II Ultrashield Plus spectrometer operating at 600 MHz. The spectra were recorded for the PGS prepolymer samples in CDCl_3_ or acetone-*d*_6_, whereas tetramethylsilane (TMS) was used as the internal standard.

#### 2.2.2 Gel Permeation Chromatography (GPC)

The PGS prepolymers were dissolved in chloroform, and solutions with a concentration 0,3% w/v were passed through a VISCOTEK VE 1122 solvent delivery system with a flow rate 1 mL/min and a temperature of 35 °C. The GPC analysis was performed using two Mixed C Styragel columns with a mixed bed (Mw = 200–2000000) and a refractive index detector. Narrow molar mass dispersity polystyrene standards from the EasiCal Preprepared Calibration Kit were used to generate the calibration curve.

#### 2.2.3 Fourier Transform Infrared Spectroscopy (FTIR)

The FTIR spectra of the obtained PGS prepolymers were acquired on Jasco FTIR-6700 spectrometer equipment with an ATR accessory. Measurements were taken in the 4000–400 cm^–1^ range, with 32 scans at a resolution of 4 cm ^–1^.

#### 2.2.4 Electrospray Mass Spectrometry (ESI-MS)

Electrospray ionization mass spectra were obtained by directly infusing the polymer samples into the ESI source of a Thermo LCQ Fleet ion-trap mass spectrometer (Thermo Fisher Scientific Inc., San Jose, CA, USA). PGS samples were diluted in a 1:1 (v/v) chloroform/methanol solution and introduced directly into the electrospray ionization source of a Thermo LCQ Fleet ion-trap mass spectrometer (Thermo Fisher Scientific, San Jose, CA, USA) operating in positive and negative ion mode. A syringe pump delivered the sample at a flow rate of 10 μL/min. The ESI source was set to a spray voltage of 4.8 kV, with the capillary temperature maintained at 200 °C. Nitrogen served as the nebulizing gas, and helium in the ion trap functioned as both the collision and damping gas. All measurements were carried out in positive and negative ion mode.

#### 2.2.5 Thermogravimetric Analysis (TGA)

A TGA/DSC1 Metler-Toledo thermal analyzer was used to evaluate the thermal stability of the obtained PGS prepolymers, while the obtained data were analyzed by a Mettler-Toledo Star System SW 9.30. The analysis was performed in the temperature range from room temperature until reaching 800 °C with a heating rate of 10 °C/min in a stream of N_2_ with a flow rate of 60 mL/min.

#### 2.2.6 Differential Scanning Calorimetry (DSC)

The thermal properties of the PGS prepolymers were analyzed using a nonmodulated differential scanning calorimeter TA-DSC Q2000. A nitrogen flow rate of 50 mL/min was maintained. The samples were heated from −70 to 80 °C, then cooled back to −70 °C, and subsequently reheated to 80 °C at a heating/cooling rate of 20 °C/min.

### 2.3 PGS Prepolymer Synthesis Methods

#### 2.3.1 Polycondensation of Glycerol and Sebacic Acid at High Temperature

The molar ratios of the starting monomers were 1.1:1 and 1.3:1 for glycerol to sebacic acid, respectively.^24^ A slight excess of glycerol was used to align its evaporation loss at such a high temperature. Glycerol and sebacic acid were heated at 170 °C in a round-bottomed flask under continuous stirring and inert gas atmosphere. The polycondensation was stopped after 3 h, and the obtained samples were stored at 5 °C.

#### 2.3.2 Traditional Polycondensation of Glycerol and Sebacic Acid under Reduced Pressure

The traditional PGS prepolymer synthesis can be divided into two steps: (1) first, where high temperature is applied and the monomers are continuously stirred under an inert gas atmosphere and (2) a second stage where the pressure is progressively lowered to remove the water byproduct. At the beginning, an equimolar amount of glycerol and sebacic acid was heated at a settled temperature (120 or 150 °C) in a round-bottom flask under continuous stirring and under a nitrogen atmosphere. After the first prepolymerization step, the pressure was progressively lowered until reaching 100 mbar (to remove the byproduct), and the reaction was continued before gelation was reached. The obtained samples were stored at 5 °C.

#### 2.3.3 Enzyme-Catalyzed Polycondensation of Glycerol and Sebacic Acid

The polycondensation reaction was conducted by heating an equimolar amount of glycerol and sebacic acid in a round-bottomed flask at a settled temperature (120 or 150 °C). The reaction mixture was continuously stirred and kept under an inert gas atmosphere. After a certain amount of time the temperature was decreased to 90 °C and 10 wt % of *Candida antarctica* lipase B, with regard to the monomers, was added into the reaction mixture. Next, the pressure was progressively lowered until it reached 100 mbar. The reaction was stopped before reaching gelation. Next, the obtained product was dissolved in CHCl_3_ and CALB was removed through filtration. After solvent evaporation, the obtained samples were vacuum-dried and stored at 5 °C.

#### 2.3.4 Enzyme-Catalyzed Polycondensation of Glycerol and Sebacic Acid in Acetone

This reaction is a modified version of the synthesis method described by Perin et al.^18^ Based on the analysis of their results, we selected acetone as the reaction medium. An equimolar amount of glycerol and sebacic acid, along with 4 Å molecular sieves (to remove the water byproduct from the reaction) and 10 wt % of *Candida antarctica* lipase B, with regard to the monomers, were added into a round-bottom flask. The resulting mixture was stirred for 48 h at 40 °C. After the reaction was completed, CALB was removed by filtration, and the resulting PGS prepolymer was dried under vacuum to remove the acetone. The obtained product was stored at 5 °C. The product was then stored at 5 °C.

#### 2.3.5 Amberlyst-15-Catalyzed Polycondensation of Glycerol and Sebacic Acid

An equimolar amount of glycerol and sebacic acid was heated at 120 °C in a round-bottom flask for 24 h with continuous stirring under an inert gas atmosphere. Next, the temperature was lowered to 90 °C and 10 wt % of dry Amberlyst-15 (A-15), with regard to the monomers, was added into the reaction mixture. The reaction was stopped after 6 h before gelation was reached. The obtained product was dissolved in CHCl_3_ and the A-15 catalyst was removed through filtration. After solvent evaporation the obtained samples were vacuum-dried and stored at 5 °C.

#### 2.3.6 The Influence of the CALB-Enzyme on the PGS Prepolymer

To evaluate the effect of the used CALB catalyst on the properties of the final PGS prepolymers, two comparative syntheses were performed under identical reaction conditions (time, temperature, and pressure). In both syntheses, equimolar amounts of glycerol and sebacic acid were used, and the initial prepolymerization step was carried out at 150 °C under an inert gas atmosphere with continuous stirring. In the second step, the temperature was decreased to 90 °C in both reactions. The 10 wt % of CALB catalyst was added to one synthesis and the reaction was carried out according to the protocol described in the Enzyme-Catalyzed Polycondensation of Glycerol and Sebacic Acid section, while the second synthesis (the control experiment) was continued without enzyme while maintaining the same temperature and reaction time. Both syntheses were stopped after 70 h. The PGS prepolymer obtained by the enzyme-catalyzed method was dissolved in chloroform, filtered, and vacuum-dried, while the control sample was analyzed as received.

## 3 Results and Discussion

### 3.1 The Influence of the Reaction Method on the GPC Results

In order to compare the influence of the selected synthesis method on the properties of the obtained PGS prepolymer, five different synthesis methods were used: (1) high temperature polycondensation, (2) traditional polycondensation under reduced pressure, (3) enzyme-catalyzed polycondensation, (4) enzyme-catalyzed polycondensation in acetone, and (5) Amberlyst-15-catalyzed polycondensation. Scheme 1 presents the general reaction scheme for PGS prepolymer synthesis through the polycondensation of glycerol with sebacic acid.

**Scheme 1:**
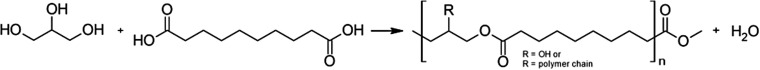
Polycondensation of Glycerol with Sebacic Acid for PGS Prepolymer Synthesis

The molar ratios of the starting monomers (glycerol to sebacic acid), the polycondensation reaction conditions, and the number-average molar masses, weight-average molar masses, and dispersities of the obtained PGS prepolymers, as determined by GPC, are listed in Table 1.

**Table 1:** Synthesis Conditions for the Conducted PGS Prepolymer Reactions^a^

sample	G:S molar ratio	temperature [°C]^a^	time [h]^b^	pressure [mbar]	*M*_n_	*M*_w_	*M*_w_/*M*_n_
Polycondensation of Glycerol and Sebacic Acid at High Temperature							
PGS1	1.1:1	170	3		700	1900	2.7
PGS2	1.3:1	170	3		800	2250	2.8
Traditional Polycondensation of Glycerol and Sebacic Acid under Reduced Pressure							
PGS3	1:1	120	(1) 24	100	900	3000	3.3
			(2) 48				
PGS4	1:1	120	(1) 24	100	ND	ND	ND
			(2) 68				
PGS5	1:1	120	(1) 7	100	900	2950	3.3
			(2) 16				
PGS6	1:1	(1) 120; 150	(1) 1; 3.5	100	ND	ND	ND
		(2) 150	(2) 24				
Enzyme-Catalyzed Polycondensation of Glycerol and Sebacic Acid							
PGS_E1	1:1	(1) 120	(1) 7	100	2100	5300	2.5
		(2) 90	(2) 72				
PGS_E2	1:1	(1) 120	(1) 24	100	2750	5200	1.9
		(2) 90	(2) 46				
PGS_E3	1:1	(1) 120; 150	(1) 1; 3.5	100	2250	6250	2.8
		(2) 90	(2) 24				
PGS_ E4	1:1	(1) 150	(1) 4.5	100	2300	4300	1.9
		(2) 90	(2) 70				
Enzyme-Catalyzed Polycondensation of Glycerol and Sebacic Acid in Acetone							
PGS_MS	1:1	40	48	-	2300	20350	8.9
Amberlyst-15-Catalyzed Polycondensation of Glycerol and Sebacic Acid							
PGS_A15	1:1	(1) 120	(1) 24	-	1150	4650	4.0
		(2) 90	(2) 6				
The Influence of the CALB-Enzyme on the PGS Prepolymer							
PGS7 control experiment without enzyme	1:1	(1) 150	(1) 4.5	100	500	2000	4.0
		(2) 90	(2) 70				
PGS_ E4	1:1	(1) 150	(1) 4.5	100	2300	4300	1.9
		(2) 90	(2) 70				

a Synthesis conditions include the glycerol (G) to sebacic acid (S) molar ratio of the starting monomers, reaction temperature, time, and assigned pressure with a summary of the GPC results: number- average molar mass (*M*_n_), weight -average molar mass (*M*_w_), and dispersity (*M*_w_/*M*_n_). ND: not determined, gelation occurred during polycondensation.

b The first subpoint (1) describes the temperature set for the initial step of the prepolymerization reaction, while the second (2) describes the temperature after applying vacuum. For the samples PGS6 and PGS_E3, in the first step (1), the reaction temperature was 120 °C for 1 h, which was then increased to 150 °C for the next 3.5 h.

c The first subpoint (1) shows the duration of the initial step of the prepolymerization reaction, while the second (2) shows the duration of the reaction after applying vacuum. For the samples PGS6 and PGS_E3, in the first step (1), the reaction temperature was 120 °C for 1 h, which was then increased to 150 °C for the next 3.5 h.

The GPC results revealed that the chosen synthesis method influences the final molecular weights and dispersities of the obtained PGS prepolymers. Polycondensation at a high temperature (170 °C) with an excess of glycerol in the starting mixture led to PGS prepolymers with the lowest molecular weights (*M*_n_ = 800 with a 30% excess of glycerol). Traditional polycondensation under reduced pressure with an equimolar amount of the starting monomers resulted in PGS prepolymers with approximately *M*_n_ = 900 and a dispersity of 3.3. It is worth noting that extending the duration of the initial prepolymerization step (see sample PGS4, Table 1) or increasing the temperature (see sample PGS6, Table 1) led to gelation, resulting in cross-linked samples and therefore limiting the increase in molar mass of the PGS prepolymer by this method. Additionally, for the samples obtained by the uncatalyzed synthesis methods (from PGS1 to PGS6 in Table 1) the GPC results clearly revealed the presence of two distinct peaks. Q. Liu et al. suggest that the first broad peak is corresponded to the sol part, and the second to sebacic acid.^25^ These authors also indicate that higher molecular weights of the PGS prepolymers result in samples with higher viscosity and therefore influence the rheological properties of the prepolymers.

While the uncatalyzed polycondensation of glycerol and sebacic acid in bulk was limited by gelation, the synthesis of PGS prepolymer using CALB and A-15 catalysts was applied. The use of CALB catalyst in the bulk reaction of glycerol and sebacic acid enabled the formation of PGS prepolymers with the highest molecular weight and lowest dispersity among all the synthesis methods applied (with *M*_n_ reaching up to 2750 and a dispersity of 1.9), whereas for the CALB-catalyzed polycondensation performed in acetone at 40 °C, a PGS prepolymer with *M*_n_ = 2300 and a very high *M*_w_/*M*_n_ value of 8.9 was obtained. The absence of gelation in the enzyme-catalyzed polycondensation aligns with findings in earlier studies.^16^ However, the Amberlyst-15 catalyst showed different behavior than the CALB enzyme, leading to the formation of PGS prepolymers with higher molecular weight than those obtained in the uncatalyzed reactions (*M*_n_ = 1150, *M*_w_ = 4.04 for PGS_A15, Table 1) but lower than those synthesized by the CALB-catalyzed method. Furthermore, gelation occurred upon extending the reaction time, thereby limiting the increase of the molecular weight of the PGS prepolymers catalyzed by A-15. Moreover, the control experiment (see PGS7, Table 1) revealed that the CALB enables the formation of PGS prepolymers with higher molecular weight and lower dispersities compared with polycondensation under the same reaction conditions without the catalyst. This confirms that this enzyme can be successfully used to catalyze the prepolymerization step in the PGS synthesis.

It is worth noting that in the above discussion we used the average molar masses of the obtained PGS samples to estimate the differences between them. However, it should be noted that the values presented in Table 1, determined by GPC using narrow polystyrene standards, may deviate from the actual values. The obtained PGS samples are partially branched with sebacic acid as the branching unit. For oligomers containing branched chains, these values may be underestimated because the more compact conformation of branched macromolecules in solution leads to a reduction in their hydrodynamic volume. Therefore, the reported molecular weights should be considered as likely underestimates.

### 3.2 Structural Characterization of the Obtained PGS Prepolymers

The application of FTIR analysis enabled precise monitoring of the polycondensation reaction progress and determination of the structure of the resulting products. Figure 1 shows the FTIR spectra of the PGS4 sample, synthesized through traditional polycondensation under reduced pressure, after different reaction times. As markers of reaction progress, the bands corresponding to the stretching of the hydroxyl group (∼3300 cm^–1^) and the carbonyl group (∼1700 cm^–1^) were used. It was observed that the broad band at ∼3300 cm^–1^ shifted toward higher wavenumbers, with a simultaneous decrease in intensity as the reaction progressed. Moreover, the sharp peak at ∼1691 cm^–1^, attributed to the carbonyl stretching of the carboxyl groups of sebacic acid, shifted to higher wavenumbers (∼1733 cm^–1^), as the reaction progressed, indicating the formation of ester groups.^18,26–28^

**Figure 1:**
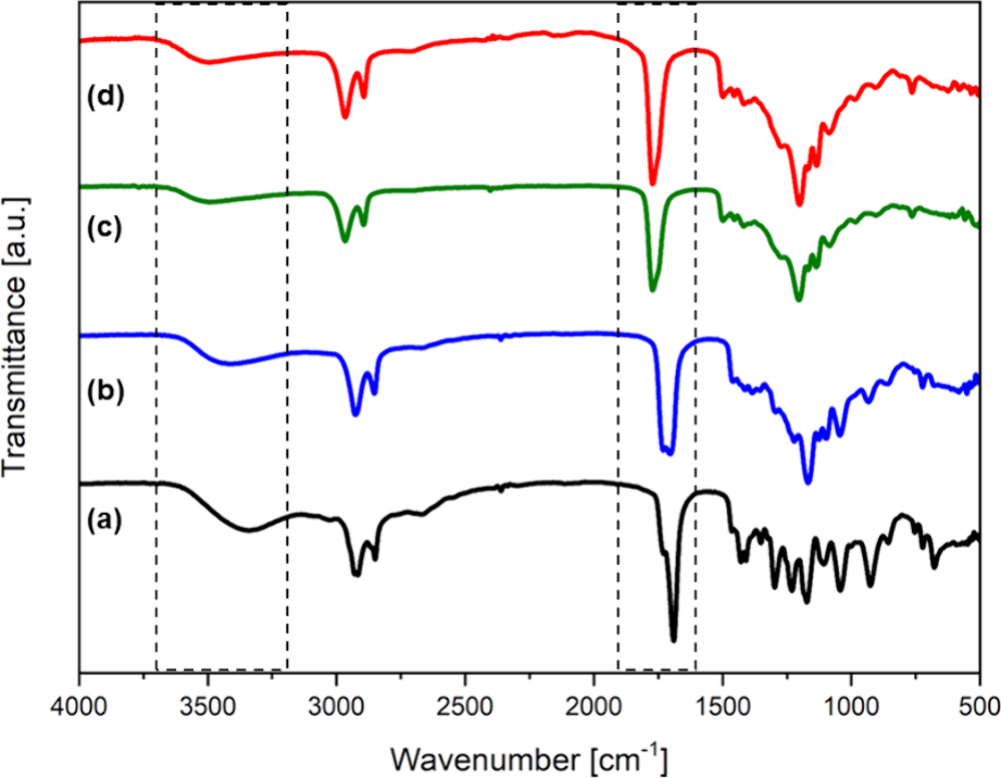
FTIR spectra of the prepolymer sample PGS4 obtained through the polycondensation under reduced pressure conducted in bulk without any catalysts after (a) 4.5 h, (b) 24 h, (c) 48 h, and (d) 68 h. The selected reaction progress markers are shown within the dashed frames.

The structures of the PGS prepolymers obtained through various synthetic methods were preliminarily characterized by using FTIR. All product samples exhibited bands in the FTIR spectra that are typical of PGS prepolymers. The characteristic signals at 2932 cm^–1^ and 2857 cm^–1^ are attributed to the asymmetric and symmetric stretching vibrations of the −CH_2_ groups. The broad band at 3300 cm^–1^ is correlated to O–H groups stretching vibrations, while the intense signal at 1730 cm^–1^ corresponds to carbonyl stretching vibrations C=O of formed ester groups. Furthermore, the characteristic sharp band at 1161 cm^–1^ may be attributed to the stretching vibration of the C–O.

An important step in this work was to identify the differences in the chemical structures of the obtained samples in order to estimate the impact of the chosen synthesis method on the chemical structure of the final PGS prepolymers. For this purpose, we conducted a detailed structural characterization using one-dimensional (1D) and two-dimensional (2D) NMR analyses and compared the results to available literature data. The ^1^H NMR spectra obtained for the all synthesized PGS prepolymers contain a characteristic region of overlapping signals between 3.4 and 4.4 ppm (Figure 2). These overlapping signals correspond to the protons of various glyceridic units formed during the polycondensation of trifunctional glycerol (G) with bifunctional sebacic acid (S) (Figure 3).

**Figure 2:**
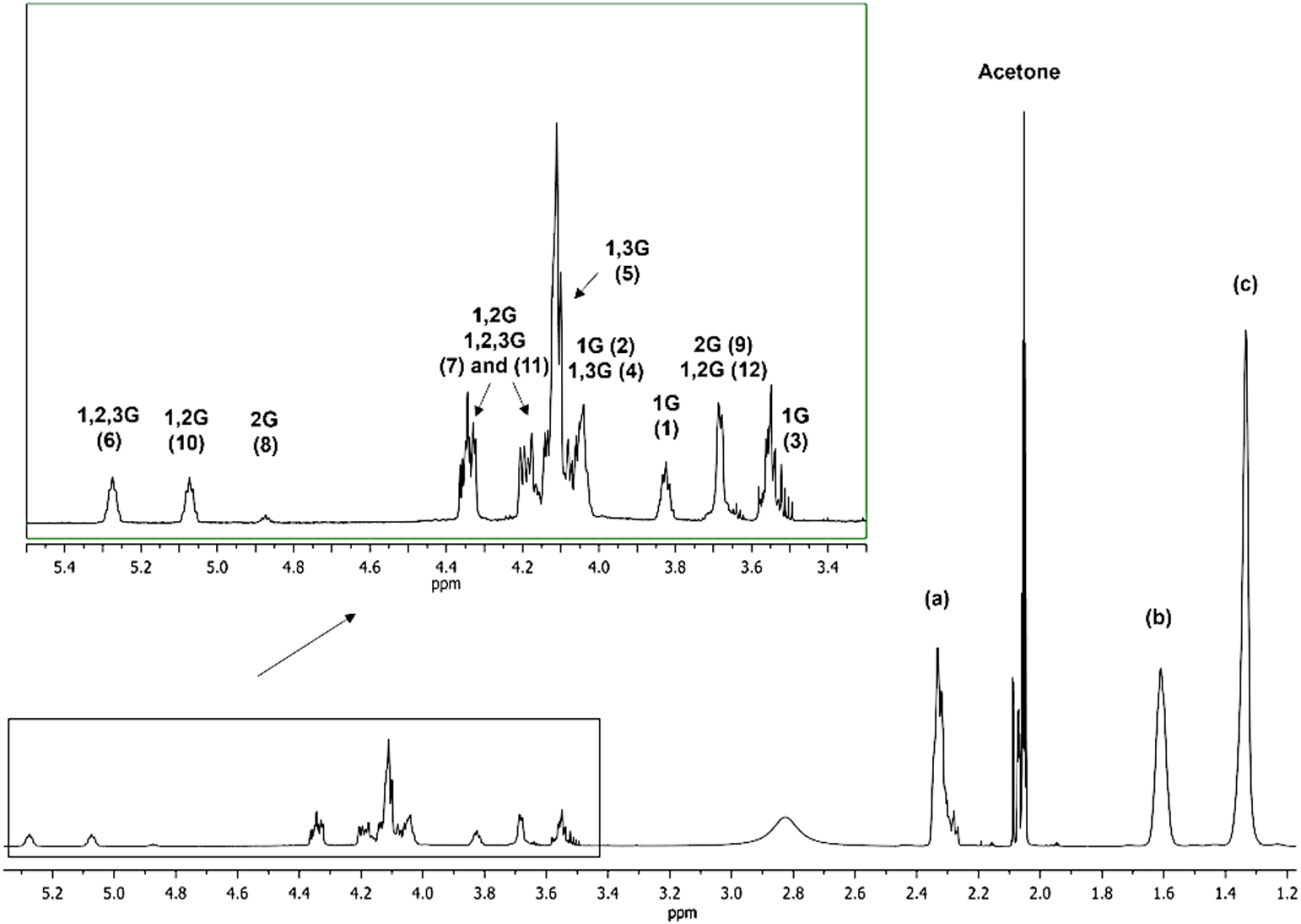
^1^H NMR spectrum of the obtained PGS prepolymer in acetone-*d*_6_ obtained through the enzyme-catalyzed reaction.

**Figure 3:**
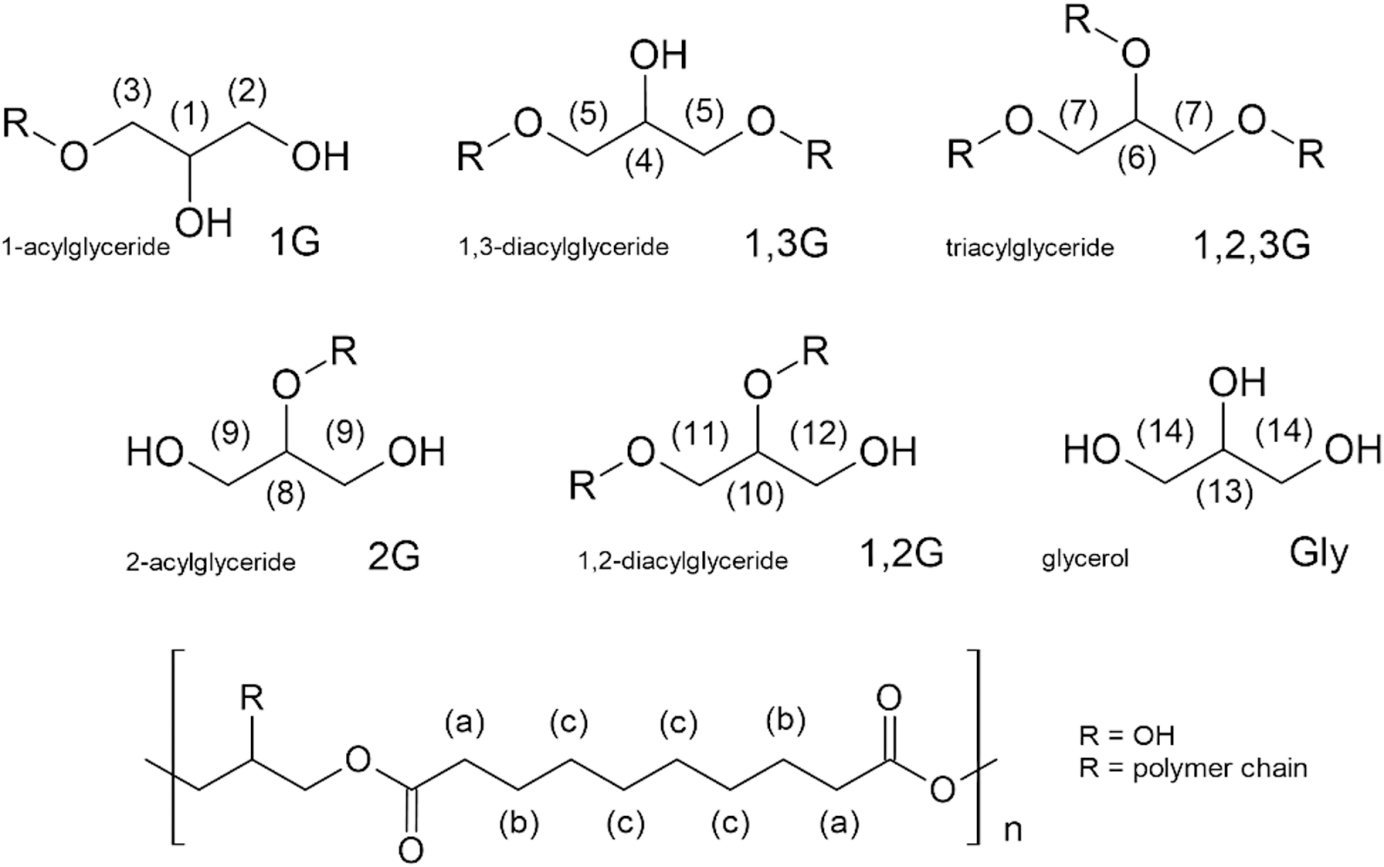
Chemical structure of PGS prepolymer and structures of various glyceridic units that can be formed during the polycondensation of glycerol with sebacic acid.

The ^1^H NMR spectrum of the analyzed PGS prepolymer obtained via the enzyme-catalyzed polycondensation revealed three specific regions: (1) the first from 1.2 to 2.4 ppm corresponding to the signals from the protons of the sebacic acid units in the prepolymer, (2) the shift region from 3.4 to 4.4 ppm correlated to the overlapping signals of the methylene protons of the glyceridic units and the signal at 3.83 ppm corresponding to the methine proton of 1-acylglyceride (1G), and (3) the signals in the region between 4.8 and 5.4 ppm from the free of overlapping signals of the methine protons of the 2G, 1,2G, and 1,2,3G units.

Usually, primary hydroxyl groups of the glycerol monomers react more quickly than secondary hydroxyl groups in the early stages of the polymerization. This leads to the appearance of the monosubstituted glyceridic unit 1-acylglyceride (1G) and as the reaction progresses, the polymer chain grows and linear 1,3-diacyloglyceridic (1,3G) units are formed. However, the less reactive secondary hydroxyl group can also react with the carboxylic groups from the sebacic acid when glycerol is less available. This may occur, for example, due to the evaporation of glycerol at high reaction temperatures or during prolonged reaction times, where the availability of glycerol decreases simply as a result of increasing chain length. As a result of this, glyceridic units with a substituted secondary hydroxyl group appear, namely, 2-acylglyceride (2G), 1,2-diacylglyceride (1,2G), and triacylglyceride (1,2,3G). Nevertheless, we expect a higher amount of the linear, primary substituted glyceridic units (such as 1G and 1,3G) compared to secondary substituted ones (2G, 1,2G, and 1,2,3G) for all PGS prepolymers. However, it should also be noted that the formation of cyclic compounds via intramolecular condensation may occur, after which the progression of the molecular chain length of the PGS prepolymer is stopped.^3^

As mentioned earlier, the assignment of signals in the ^1^H NMR spectrum is challenging due to the overlapping regions associated with the presence of various glyceridic units and unreacted glycerol monomers. This issue can be overcome by performing ^13^C NMR analysis in which the signals from the glyceridic units are well-resolved and can be assigned to the specific carbon atoms of each glyceridic unit or the glycerol monomer. Furthermore, the two-dimensional (2D) NMR analyses enable the detailed assignment of signals observed in the ^1^H NMR spectrum. Figures 2 and 4–6 show the ^1^H NMR, ^13^C NMR, ^1^H–^1^H COSY, and ^1^H–^13^C HSQC spectra in acetone-*d*_6_ of the PGS prepolymer obtained via the CALB-catalyzed polycondensation after 24 h of reaction.

**Figure 4:**
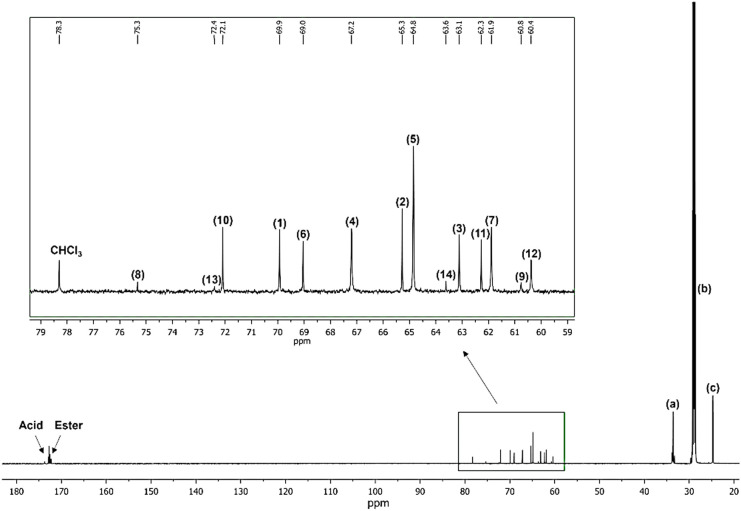
^13^C NMR of the obtained PGS prepolymer in acetone-*d*_6_ obtained through the enzyme-catalyzed reaction.

The signals in the shift region 20–35 ppm marked as a, b, and c refer to the carbons of sebacate units in the PGS prepolymer (Figure 4). The enlarged region of the ^13^C NMR spectrum shows signals corresponding to the 14 types of carbons assigned to the various glyceridic units or glycerol (Figure 4). The peak present at 78.3 ppm is attributed to the chloroform solvent residue, which was used after the polycondensation to separate the CALB-enzyme from the reaction medium. The obtained spectrum shows great similarity to the ones reported in literature.^18,22,28,29^

Based on the assignment of the signals in the ^13^C NMR spectrum (Figure 4), we can relate the carbons with their protons in the PGS prepolymer. Figure 5 shows the enlargement of the region in the range 3.0–5.5 ppm in which we have the overlapping signals from the glyceridic units. It is possible to connect almost each carbon of the glyceridic units with their protons except the unreacted glycerol or the methine proton marked as (8) of the 2-acylglyceride (2G) unit, which may be related to too low content of such structures in the analyzed PGS prepolymer sample.

**Figure 5:**
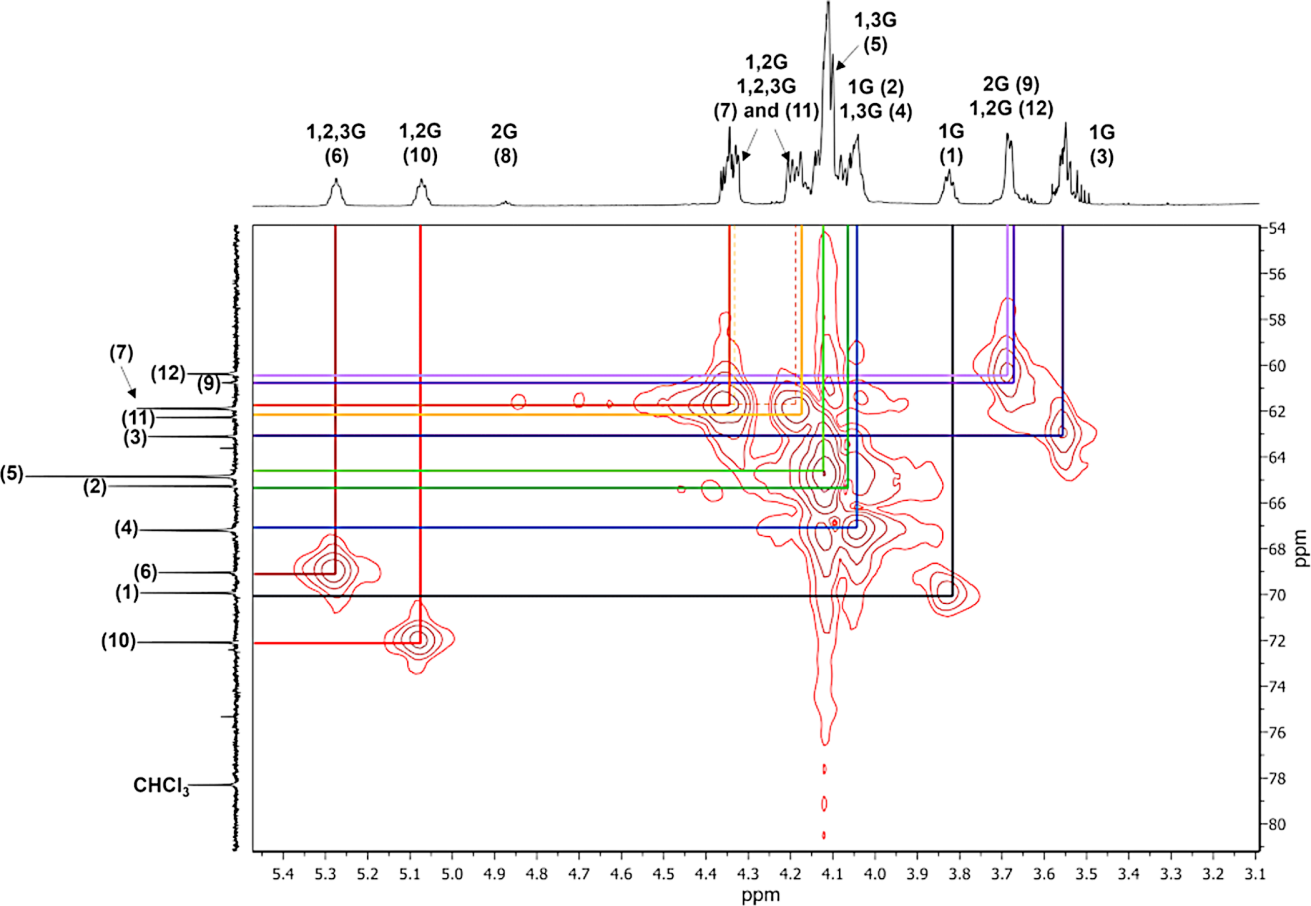
^1^H–^13^C NMR spectrum of the PGS prepolymer obtained through the enzyme-catalyzed reaction.

The proton–proton NMR spectroscopy analysis revealed signals (free from overlapping) of the methine protons of the 1G, 2G, 1,2G, and 1,2,3G glyceridic units (Figure 6). Additionally, the signals from the 1,3G glyceridic unit are present.

**Figure 6:**
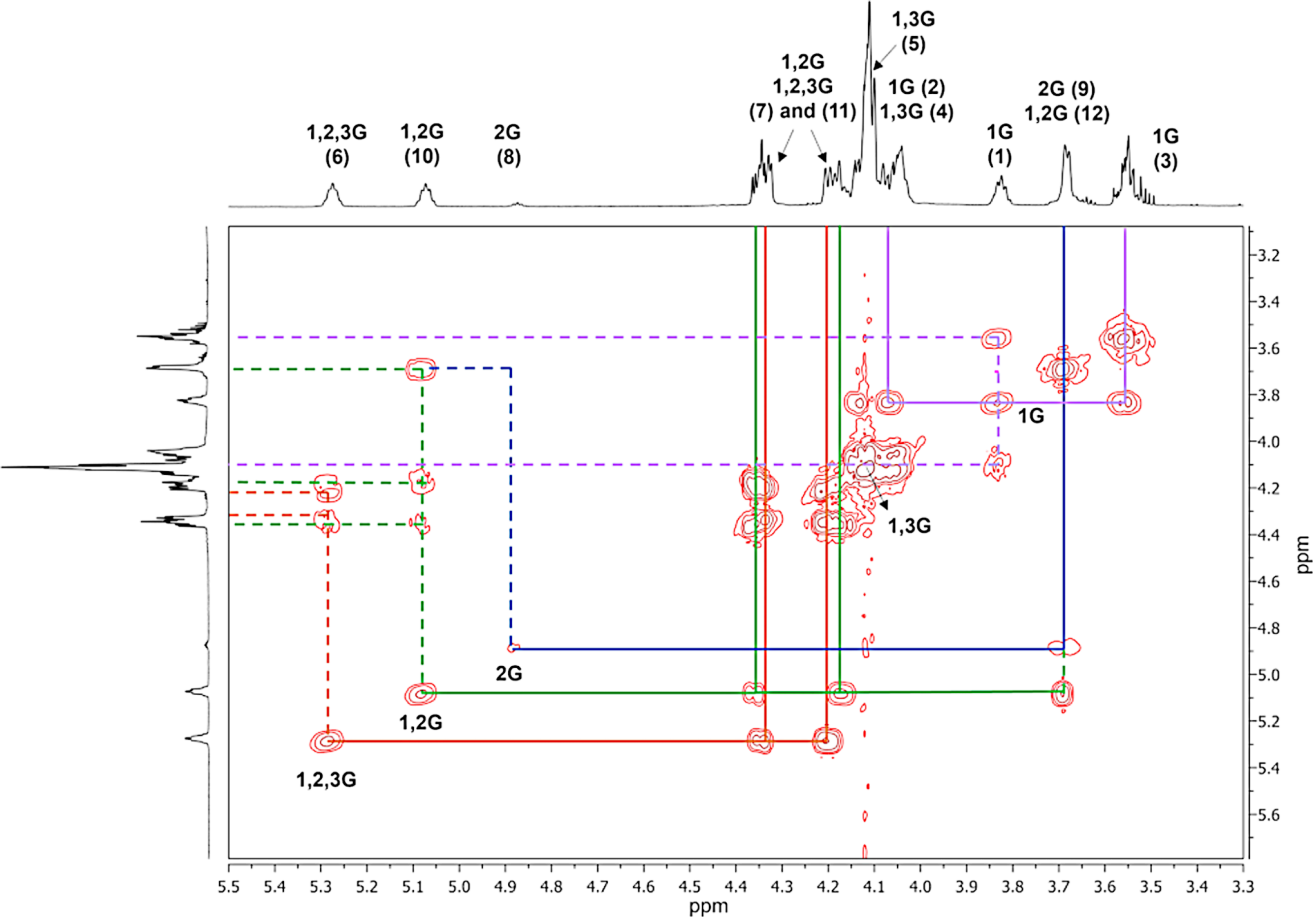
^1^H–^1^H NMR spectrum in acetone-*d*_6_ of the PGS prepolymer obtained through an enzyme-catalyzed reaction.

To summarize this part of the research, the use of ^13^C NMR spectroscopy and two-dimensional (2D) NMR analysis enabled a detailed assignment of the signals observed in the ^1^H NMR spectra of the obtained PGS prepolymers, allowing for the determination of their detailed chemical structure. In the analyzed PGS prepolymer samples, the signals corresponding to the protons of unreacted glycerol monomers were not assigned, as they were not observed in the ^1^H–^13^C NMR correlation spectra, even though their signal assignments have been described in the literature.^18^

### 3.3 Determination of Glyceridic Unit Content (in mol %) and Unit Composition

To examine how the synthesis conditions affect the structure of the obtained PGS prepolymer samples, the proportions of the individual glyceridic units (in mol %) were determined based on the analysis of their ^1^H NMR spectra (see Figures S1, S2, S3, and S4 in Supporting Information), using the method described by Perin et al.^18^ Additionally, the molar ratio of glycerol to sebacic acid in the prepolymers was estimated using the formula proposed by Liu et al.^30^ The results of these analyses are summarized in Table 2.

**Table 2:** Content of Each Glyceridic Unit (in mol %) in the Obtained PGS Prepolymer Samples Determined Based on ^1^H NMR Spectra Recorded in CDCl_3_ ^a^ and the Monomer-Derived Unit Composition of Glycerol (G) to Sebacyl (S) in the PGS Prepolymers

	content of each glyceridic unit in mol %					monomer-derived unit composition
sample	1-acylglyceride (1G)	2-acylglyceride (2G)	1,2-acylglyceride (1,2G)	triacylglyceride (1,2,3G)	1,3-acylglyceride (1,3G)	G:S^a^
Polycondensation of Glycerol and Sebacic Acid at High Temperature						
PGS1	7	1	12	23	58	0.68:1
PGS2	9	1	12	17	60	0.65:1
Traditional Polycondensation of Glycerol and Sebacic Acid under Reduced Pressure						
PGS3	20	2	15	15	48	0.99:1
PGS4	ND					
PGS5	21	2	8	13	56	0.92:1
PGS6	ND					
Enzyme-Catalyzed Polycondensation of Glycerol and Sebacic Acid						
PGS_E1	15	3	12	20	50	0.91:1
PGS_E2	12	1	15	24	48	0.85:1
PGS_E3	21	3	15	21	40	0.93:1
PGS_E4	16	2	17	24	40	0.92:1
Enzyme-Catalyzed Polycondensation of Glycerol and Sebacic Acid in Acetone						
PGS_MS	22	5	16	23	34	0.99:1
Amberlyst-15-Catalyzed Polycondensation of Glycerol and Sebacic Acid						
PGS_A15	18	3	15	15	50	0.89:1
The Influence of the CALB-Enzyme on the PGS Prepolymer						
PGS7^b^	4	5	17	15	58	1.01:1
PGS_ E4	16	2	17	24	40	0.92:1

a The formula used to estimate the actual molar ratio of glycerol (G) to sebacyl (S) units in the obtained PGS prepolymers is the following. Actual molar ratio_(G)/(S)_: *F* = $\frac{16{I\delta }_{\left(d,e,f\right)}}{5{I\delta }_{\left(a,b,c\right)}},$ where δ_a_ = 1.237–1.287; δ_b_ = 1.608; δ_c_ = 2.287–2.346; δ_d_ = 3.577–3.927; δ_e_ = 4.079–4.297; δ_f_ = 4.920 + 5.077 + 5.248; *I* = the area of peaks; δ = chemical shift (ppm); a, b, c, d, e, f = category of hydrogen protons; (G) = glycerol; (S) = sebacic acid.

b Control experiment without enzyme.

Based on the results presented in Table 1 and Table 2, we can draw conclusions regarding the influence of the synthesis method on the chemical structure and content of each glyceridic unit in the PGS prepolymer. Regarding the monomer-derived unit composition, we can assume that higher polycondensation temperatures appear to disrupt the equimolar ratio of the monomers. Therefore, it is important to note that the synthesis method influences the glycerol to sebacyl unit composition in PGS prepolymers, as determined by ^1^H NMR.

In the case of polycondensation reactions carried out at very high temperatures (170 °C) in a relatively short time (for 3 h), the formation of predominantly linear 1,3G glyceridic units was observed. However, it should be noted that an excess of the glycerol monomer is required to compensate for the glycerol loss at high reaction temperatures. This reaction pathway enables the rapid formation of linear glyceridic units with a relatively low molecular weight and high dispersity. Similarly, polycondensation under reduced pressure enables the formation of predominantly linear oligomers (1,3G units constitute up to 58 mol % of the PGS prepolymer) with small amounts of branched species. In this case as well, the resulting PGS prepolymer is characterized by relatively low average molar masses and a relatively high dispersity index. Additionally, these reactions are both energy- and time-consuming, and control over the gelation process is limited and difficult to predict. To address these limitations, catalytic approaches have been employed in the synthesis of PGS prepolymers. During the polycondensation reaction catalyzed by *Candida antarctica* lipase B, a high amount of linear glyceridic units is formed, yet an unexpected increase in branched glyceridic units is also observed. It was assumed that during the CALB-catalyzed reactions, mainly linear glyceridic units would form due to the *sn*-1,3 regiospecific character of the CALB enzyme, which prefers the primary hydroxyl groups of glycerol monomers for esterification catalysis. However, acyl migration can lead to the formation of branched species (such as 1,2,3G), as esterification may also occur at the secondary hydroxyl group.^31^ This may explain the slightly increased amount of branched (cross-linked) structures (over 20%, as determined by ^1^H NMR) in the PGS prepolymer samples obtained via enzyme-catalyzed polycondensation. Moreover, it should be noted that PGS prepolymers with higher average molar masses and lower dispersity compared to those obtained through the uncatalyzed polycondensation were synthesized using the enzyme-catalyzed method. Additionally, gelation did not occur, which enabled precise control of the reaction process. Interestingly, the CALB-catalyzed polycondensation in acetone (PGS_MS), with the addition of molecular sieves, resulted in almost evenly distributed glyceridic units in the PGS prepolymer. Furthermore, the obtained product was characterized by a relatively high *M*_w_ and broad dispersity index, distinguishing it significantly from other PGS samples synthesized via bulk polycondensation. Different results were obtained when Amberlyst-15 was used as the catalyst for the polycondensation of glycerol and sebacic acid. This synthetic approach enabled the formation of a PGS prepolymer (sample PGS_A15) in a relatively short time (24 h for the first prepolymerization step, followed by 6 h for the second step of the catalyzed polycondensation). The resulting product contained a high amount of linear glyceridic units, where 1,3G units consisted of half of the PGS prepolymer product. However, with an extended reaction time, gelation occurred, distinguishing this method from the CALB-catalyzed approach.

Additionally, to evaluate the effect of the CALB catalyst on the chemical structure of the final PGS prepolymers, two comparative polycondensation reactions of glycerol with sebacic acid were carried out under identical conditions (time and temperature) with and without the catalyst. The ^1^H NMR analysis revealed that, after 70 h, the control experiment (without the catalyst) resulted in a higher content of linear glyceridic units (1,3G and 1G) compared to the enzyme catalyzed reaction. However, it should be noted that the sample PGS7 was characterized by significantly lower average molecular masses and a higher dispersity index than the enzyme-catalyzed product. These results suggest that the use of the CALB enzyme in the PGS prepolymer synthesis facilitates polymer chain growth, leading to the formation of prepolymers with higher molecular weights. Analysis of the results presented in Tables 1 and 2 confirmed the influence of the synthesis methods on the chemical structure and content of individual glyceride units in the PGS prepolymers. All obtained PGS prepolymers were shown to be partially branched with sebacic acid as the branching agent. The highest degree of branching was observed for PGS obtained by enzymatic catalysis, which, according to NMR results Table 2, exhibits a slightly higher degree of branching than PGS synthesized nonenzymatically. Therefore, it can be concluded that the molecular weight values of the obtained PGS given in Table 1 are probably underestimated, especially for PGS obtained by enzymatic catalysis.

### 3.4 ESI-MS Analysis of PGS Prepolymers for Structural Characterization on the Molecular Level

Electrospray ionization mass spectrometry (ESI-MS) was used to investigate the chemical structure of poly­(glycerol sebacate) (PGS) prepolymer samples synthesized under different reaction conditions.

The mild ionization conditions of the ESI source enabled the detection of low molecular weight oligomers with minimal fragmentation. The resulting +ESI mass spectra revealed complex mixtures of oligomeric species predominantly observed as sodium adducts [M + Na]^+^.

In the enlarged section of the spectrum (*m*/*z* 450–1350), a regular series of peaks appeared at *m*/*z* 483, 557, 631, 741, 815, 889, 999, 1073, 1257, and 1331 corresponding to oligomers composed of approximately one to five glyceryl-sebacyl (GS) repeating units (Figure 7). These were assigned to oligomers of the general formulas: [G­(SG)_*n*_]^+^, [(SG)_*n*_]^+^, and [S­(GS)_*n*_]^+^, with alternating glycerol and sebacic acid units and reflecting alternative end-group compositions. The consistent ∼258 Da spacing between adjacent peaks strongly supports a modular GS-based structure (C_13_H_22_O_5_, 258.1467 Da), which is typical for step-growth polycondensation.

**Figure 7:**
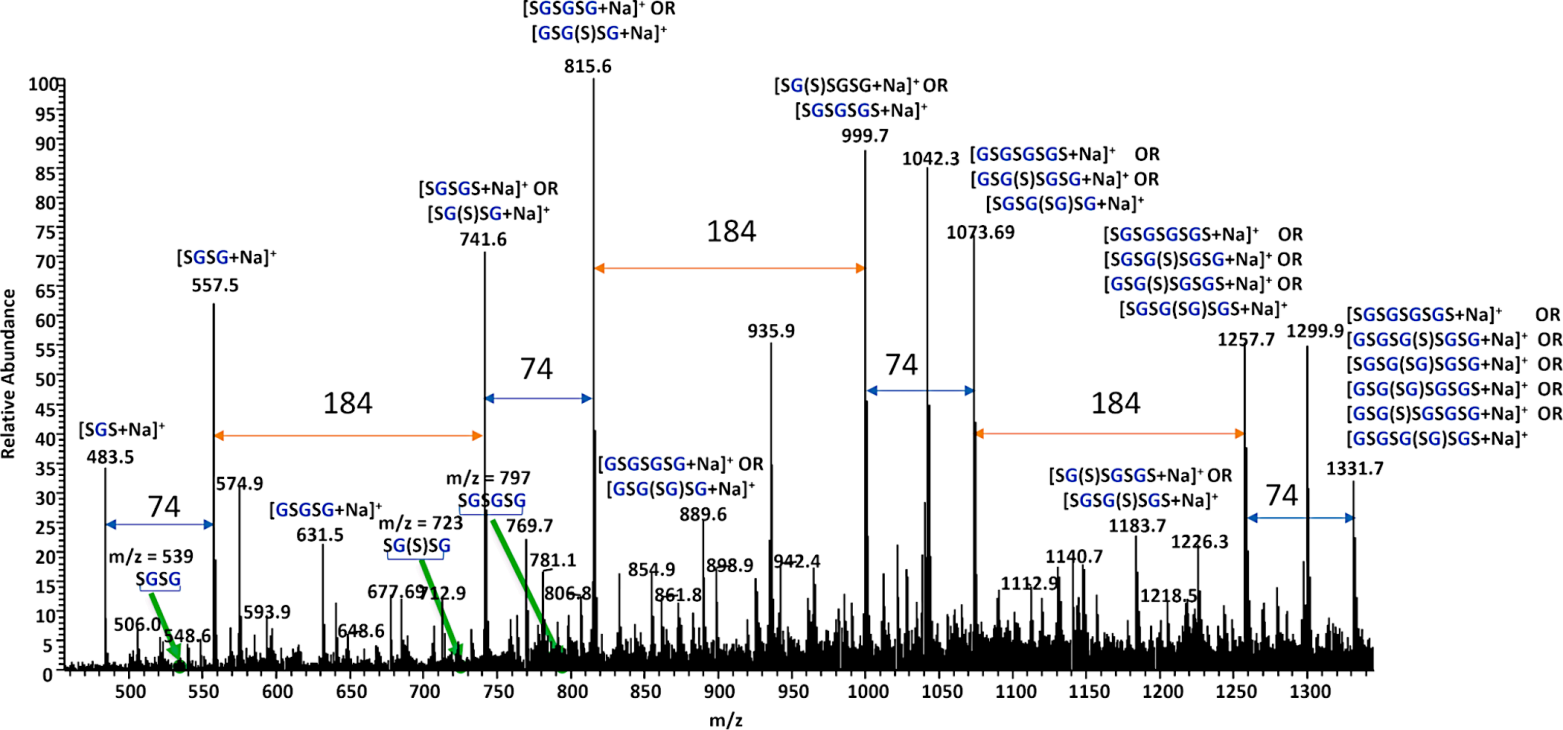
Positive ESI mass spectrum (+ESI) of the PGS prepolymer (sample PGS_E4, Table 1) representing sodium-adducts of oligomer series based on glyceryl-sebacyl repeating units.

For the ion observed at *m*/*z* 741, several possible structural isomers were proposed based on the expected combinations of glycerol and sebacic acid units. As shown in Scheme 2, these include both linear and branched isomeric structures that could correspond to singly charged sodium adduct ion [M + Na]^+^ at *m*/*z* 741. While MS cannot distinguish among isomers of the same formula, their inclusion reflects the diversity of topologies that may arise during synthesis and supports the coexistence of linear and branched architectures. Notably, the detection of higher-mass species (e.g., *m*/*z* 1257 and 1331) and deviations from the regular pattern are likely due to secondary esterification of glycerol’s hydroxyl groups, a process that favors branching, especially under prolonged reaction times or elevated temperatures.^22,29^

**Scheme 2:**
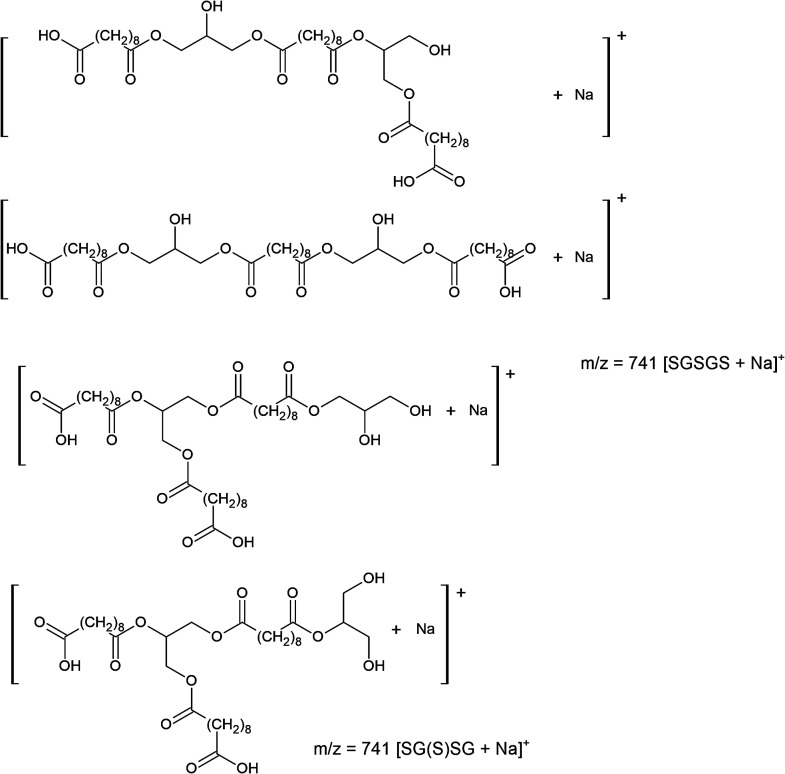
Proposed Chemical Structures of Glyceryl-Sebacyl Oligomers Corresponding to the Ion at *m/z* 741 Observed in the + ESI Mass Spectrum of the PGS Prepolymer (Sample PGS_E4, Table 1)­^a^

a The structures represent possible linear and branched isomers formed by different arrangements of glycerol and sebacic acid units.

Overall, the positive ESI data suggest that the prepolymer consists predominantly of linear oligomers with a minor population of branched species. The observed mass distribution strongly supports a modular GS-based structure typical of step-growth polymerization.

Additional analyses in negative ion mode (−ESI) further supported these findings, showing deprotonated species [M – H]^−^ corresponding to oligomers with terminal carboxyl groups (Figure 8). As shown in Figure 8, the negative −ESI mass spectrum of the PGS prepolymer displays a regular series of peaks from *m*/*z* 459 to 1307. These signals were assigned to structures composed of one to five glyceryl-sebacyl (GS) repeating units. The most intense peak at *m*/*z* 717 corresponds to [SGSGS – H]^−^, indicating the predominance of trimers in the mixture. Additional signals at *m*/*z* 459, 533, 975, 1049, 1233, and 1307 were also observed, representing both shorter and longer oligomers with decreasing abundance. The consistent ∼258 Da mass spacing between adjacent peaks clearly shows sequential GS unit addition, characteristic for the stepgrowth condensation. It should be noted that −ESI preferentially detects oligomers terminated with carboxylic acid groups, whereas species with hydroxyl end groups may be underrepresented due to their lower ionization efficiency under these conditions.^29^ Taken together, these results provide valuable structural insights that go beyond what can typically be inferred from standard techniques such as NMR or FTIR. By enabling the detection of individual oligomeric species and their architectures, ESI-MS proves to be a powerful tool for evaluation of the complex molecular composition of PGS prepolymers.

**Figure 8:**
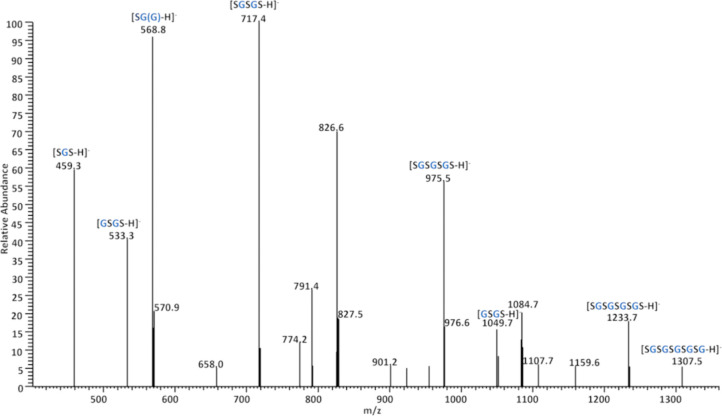
Negative ESI mass spectrum (−ESI) of the PGS prepolymer (sample PGS_E4, Table 1) indicates the presence of deprotonated oligomers with terminal carboxyl groups.

### 3.5 The Impact of the Applied Synthesis Method on the Thermal Properties of the Obtained PGS Prepolymers

The thermal properties of the obtained PGS prepolymers were determined by DSC (Figure 9a–c) and TGA (Figure 10). Table 3 presents the results of DSC analyses of samples PGS1, PGS3, PGS_E4, PGS_MS, and PGS_A15, obtained by using each of the five tested synthesis methods.

**Table 3:** Thermal Characterization of the PGS Prepolymers Obtained by Various Synthesis Methods^a^

sample	*T*_m_ [°C], I heating scan	Δ*H*_m_ [J/g], I heating scan	*T*_g_ [°C]	*T*_m_ [°C], II heating scan	Δ*H*_m_ [J/g], II heating scan	*T*_c_ [°C]	Δ*H*_c_ [J/g]
PGS1	9.51/59.36	27.68/32.29	–18.99	3.00/23.24/43.11/57.93	26.98/6.16/7.14/5.77	–20.60/–14.75	31.35
PGS3	7.65/35.88	41.08	–13.87	9.89/22.62	42.13	–16.17	39.41
PGS_E4	4.83/34.67	51.06	–21.24	7.58/21.18	26.33	–23.57	27.28
PGS_MS	7.66/42.38	21.12/8.40	–18.02	8.97/22.51	26.58	–19.48	30.84
PGS_A15	6.98/42.64	19.04/37.51	–14.22	13.44/34.77	43.64	–12.05	43.87

a *T*_g_, glass transition temperature; *T*_m_, melting temperature; *T*_c_, crystallization temperature; Δ*H*_c_, crystallization enthalpy; and Δ*H*_m_, melting enthalpy determined by DSC.

**Figure 9:**
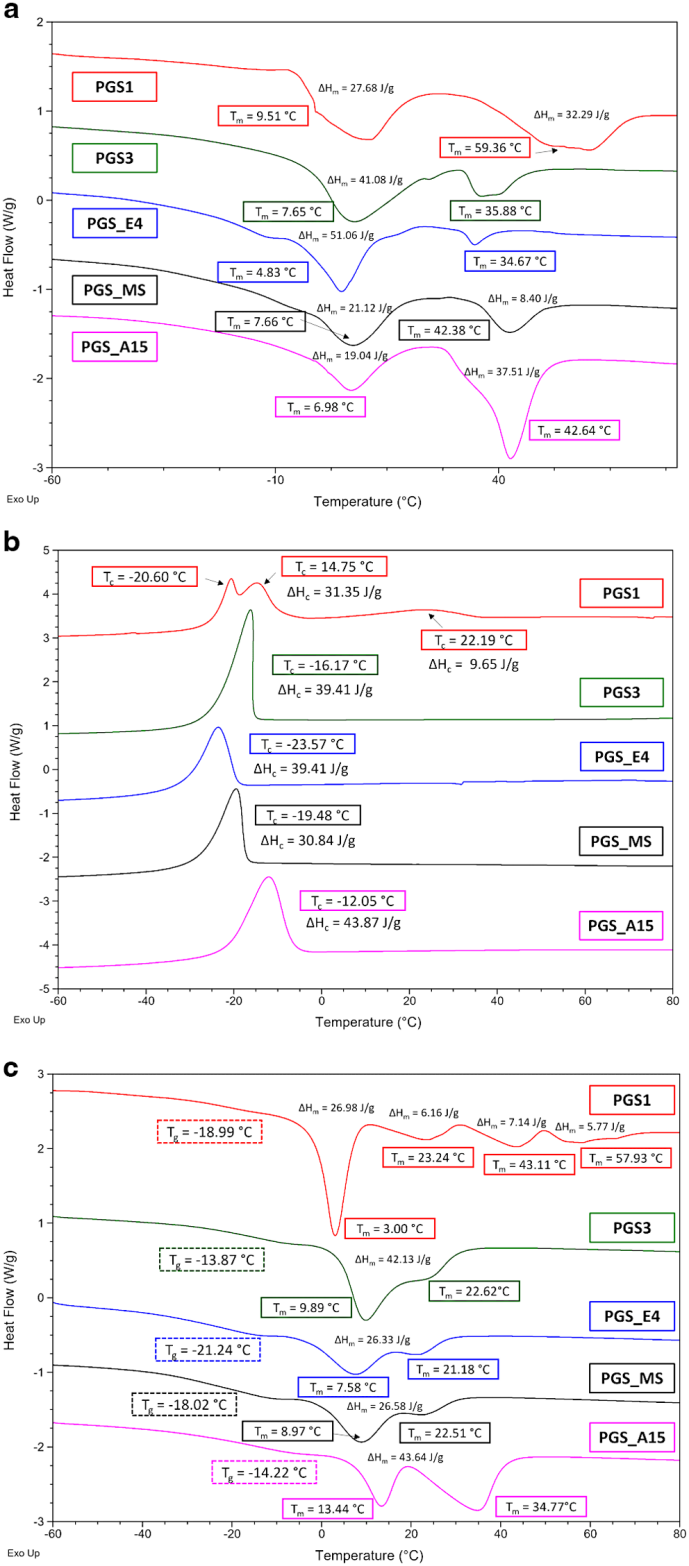
DSC thermograms of selected PGS prepolymers obtained by various polycondensation methods: (a) first heating scan, (b) cooling scan, (c) second heating scan.

**Figure 10:**
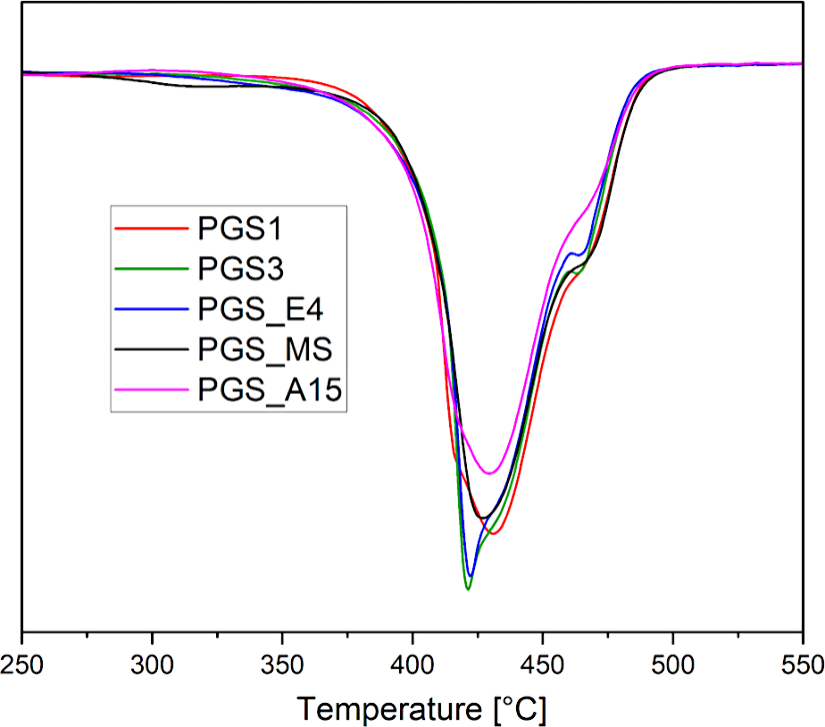
First-order derivative (DTG) curves of thermal decomposition (TGA) of selected PGS prepolymers obtained by various polycondensation methods.

The DSC results revealed differences in *T*_*g*_ among the obtained PGS prepolymers (Figure 9c), which may be attributed to varying degrees of branching and/or cross-linking. It is known that the glass transition (*T*_g_), crystallization (*T*_c_), and melting (*T*_m_) temperatures are strongly influenced by the degree of branching, cross-linking, and the monomer content in the PGS prepolymer. It should be noted that although the first step of the prepolymerization between glycerol and sebacic acid mainly forms linear oligomers, branched species are also observed (see Table 2).

For branched polymers, two opposing effects are observed in the DSC thermograms. The first is that cross-linking leads to a reduction of segment mobility, resulting in an increased glass transition temperature (*T*_g_). The second effect lowers the *T*_g_ due to an increase in the number of end groups and free volume.^32^ Some authors suggested that the lowering of *T*_g_ is a consequence of increased branching, which enhances either the flexibility or the free volume of the polymer backbone. Additionally, an important point is that branched polyesters are characterized by lower crystallization temperatures (*T*_c_) and it is assumed that a higher degree of branching can suppress crystallization as a result of steric hindrance.^33^ This is consistent with the obtained ^1^H NMR and DSC results, where for the samples with the increased content in branched glyceridic 1,2,3G units (see Table 2) it correlates with a decrease in the glass transition temperature (*T*_g_) and crystallization temperature (*T*_c_) (see Table 3, Figure 9b,c). For example, PGS3 has a *T*_g_ of −13.87 °C and *T*_c_ of −16.17 °C with a 1,2,3G content of 15%, while PGS_E4, with a *T*_g_ of −21.24 °C and *T*_c_ of −23.57 °C, exhibits a higher 1,2,3G content of 24%. Similar observations were made for poly­(glycerol adipate), which showed a decrease in *T*_g_ with the increase of branching.^34^

The first heating cycle revealed the melting transitions of the PGS prepolymers in their as-received state. It is worth noting that Lee et al. reported two melting temperatures (*T*_m_) for the PGS prepolymer at 10.7 and 27.8 °C,^32^ while Perin et al. observed similar values at 9 and 24 °C during the second heating scan.^33^ In this study, two comparable *T*_m_ values were observed in the second heating cycle for almost all of the analyzed samples (see Table 3). An exception was PGS1, which exhibited four distinct melting transitions during the second heating as well as three crystallization temperatures (*T*_c_), whereas the other samples showed only one crystallization temperature. Interestingly, de Oliveira et al. also observed additional endothermic peaks in one of their DSC heating curves for the PGS prepolymer, which they attributed to the presence of a higher content of unreacted oligomers and monomers.^35^ They suggested that the first endothermic peak corresponds to less perfect crystals, while the others correspond to more perfect crystalline domains present in smaller quantities.^34^ It is also worth noting that the two samples, PGS_E4 and PGS_MS, were synthesized by using CALB, and their thermograms exhibit very similar profiles.

For thermal stability analysis of the obtained PGS prepolymers, thermogravimetric analysis (TGA) was applied. The resulting Derivative Thermogravimetric (DTG) curves indicate that the PGS3 and PGS_E4 prepolymers exhibit two degradation steps, with the first occurring around 420 °C and the second near 465 °C. Overall, all PGS prepolymers demonstrate high thermal stability, with decomposition in a similar temperature range (*T*_*max*_) from 420 to 431 °C (Figure 10).

## 4 Conclusions

This work presents five distinct synthetic approaches for the preparation of poly­(glycerol sebacate) (PGS) prepolymers through the polycondensation of glycerol with sebacic acid. For this purpose, the following methods were applied: polycondensation at high temperatures, polycondensation under reduced pressure, enzyme-catalyzed polycondensation, CALB-catalyzed polycondensation in acetone, and Amberlyst-15-catalyzed polycondensation. To monitor the synthesis progress, FTIR analysis was used. The characteristic band corresponding to the carbonyl groups of sebacic acid shifted toward higher wavenumbers, from ∼1691 cm^–1^ to ∼1733 cm^–1^, confirming the formation of ester bonds during the polycondensation reactions. GPC analysis confirmed that the highest number-average molar masses (*M*_n_ up to 2750) and the lowest dispersity (*M*_w_/*M*_n_ = 1.9) were obtained for samples synthesized via the enzymatically catalyzed reactions. In comparison, syntheses conducted at high temperatures and under reduced pressure resulted in PGS prepolymers with lower molecular weights and higher dispersities. Additionally, these reaction conditions led to gelation in several samples upon a prolonged reaction time and increased temperature. In the case of the CALB-catalyzed reaction, no gelation was observed, indicating better control over the reaction process. The application of NMR spectroscopy (^1^H, ^13^C, and 2D NMR) enabled precise analysis of the chemical structure of the obtained PGS prepolymers. The presence of various glyceridic units (1G, 2G, 1,2G, 1,3G, and 1,2,3G) in the PGS prepolymer samples was confirmed, and their relative amounts were found to depend on the applied synthesis method. It was concluded that the number of linear and branched units varies depending on the applied synthesis method. NMR analysis played a key role in understanding the polycondensation of glycerol with sebacic acid and in revealing the influence of the reaction method on the final chemical structure and composition of the PGS prepolymers. ESI-MS analysis enabled detailed insight into the molecular architecture of the prepolymers, confirming the presence of linear and branched PGS-based oligomers and supporting findings from NMR analysis. It was concluded that as the oligomer chain length increased, the number of branches and possible structural variations also grew. The analyses conducted using DSC and TGA showed that the chosen reaction method has an impact on the thermal properties of the obtained PGS prepolymers. Samples containing more branched units exhibited lower glass transition temperatures (*T*_*g*_), which can be attributed to either enhanced flexibility or the free volume of the polymer backbone. Additionally, the decreased crystallization temperatures (*T*_c_) suggest that crystallization could be suppressed due to steric hindrance. The TGA results revealed that all prepolymers demonstrated high thermal stability (*T*_max_ in the range of 420–431 °C). The results of the conducted studies clearly confirm that the chosen reaction method has a significant impact on the chemical structure and properties of the PGS prepolymers. Enzymatic synthesis using the CALB enzyme as a catalyst provides better control over the synthesis, enabling the synthesis of PGS prepolymers with higher homogeneity, increased molecular weight, and suppressed undesirable gelation. In comparison with classical synthesis methods, the enzyme-catalyzed approach is characterized by higher efficiency and greater predictability. Obtaining a well-defined PGS prepolymer is crucial for its further application, especially in the design of controlled drug delivery systems for bioactive substances intended for use in cosmetology and medicine. This constitutes the primary objective of our future research.

## Supplementary Material



## Notes

^1^H NMR spectra of all synthesized PGS prepolymers, with selected regions of the spectra enlarged to display proton signals of individual glyceridic and sebacic units (PDF)
