# Supplementary material for: Poly(glycerol sebacate): A Comparative Study of Various Synthesis Methods
Source: Biomacromolecules. 2025 Oct 4;26(11):8146–59. doi: 10.1021/acs.biomac.5c01548 (PMC12606642; doi:10.1021/acs.biomac.5c01548)
Supplement: Supplementary file 1 [file bm5c01548_si_001.pdf]

Supporting Information for

Poly(Glycerol Sebacate): A Comparative Study of Various Synthesis Methods

*Silke Andrä-Żmuda, Paweł Chaber, Magdalena Martinka Maksymiak, Marta Musioł, Grażyna Adamus**

Centre of Polymer and Carbon Materials, Polish Academy of Sciences, 34, M. Curie-Skłodowska Street, 41-819 Zabrze, Poland

Supporting information contains 3 pages, including 4 Figures.

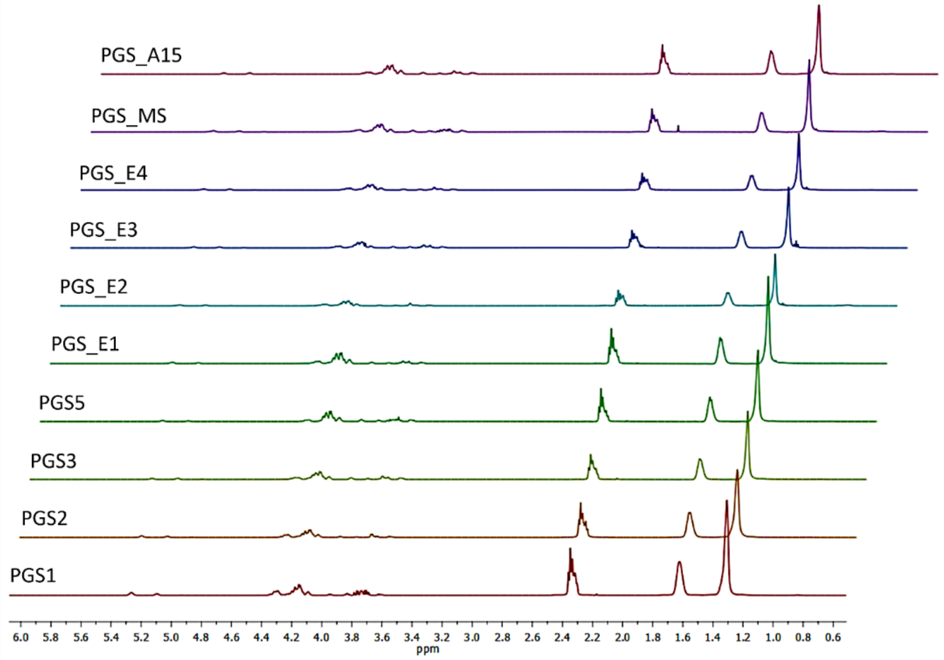

Figure S1. ^{1}H NMR spectra in $CDCl_3$ of PGS prepolymers obtained through different synthetic methods.

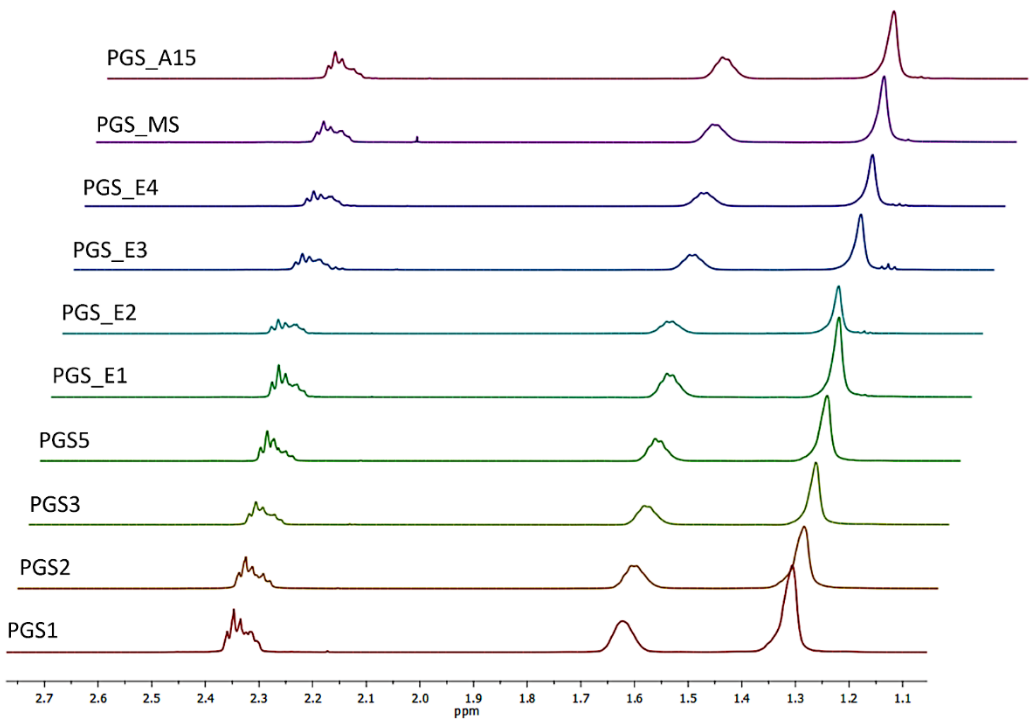

Figure S2. ^{1}H NMR spectra of PGS prepolymers in $CDCl_3$ obtained through different synthetic methods, enlarged region of the signals from the protons of the sebacic acid units in the prepolymers.

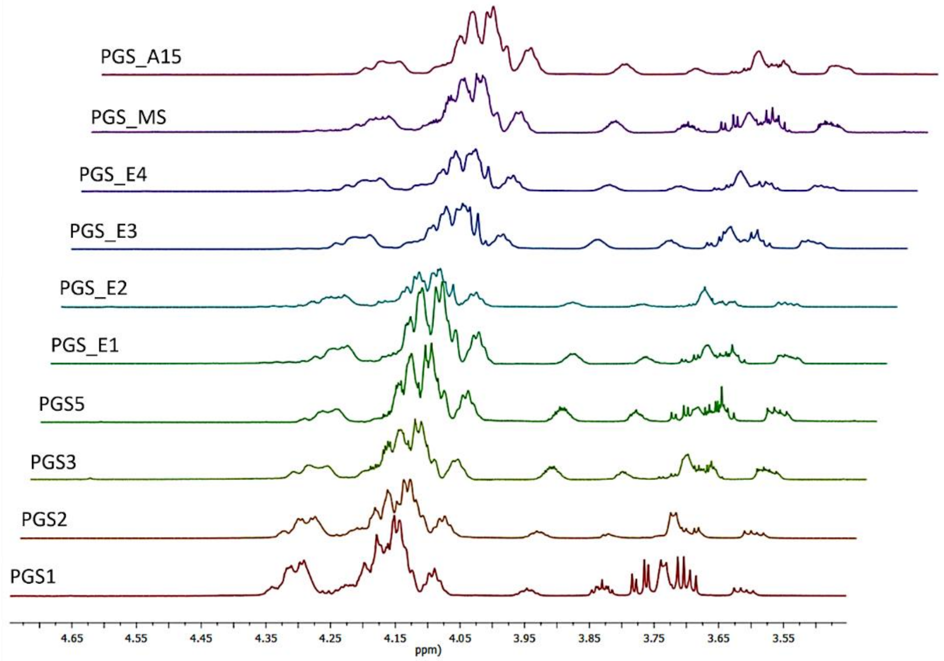

Figure S3. ^{1}H NMR spectra of PGS prepolymers in $CDCl_3$ obtained through different synthetic methods, enlarged region of the signals from the overlapping glyceridic units.

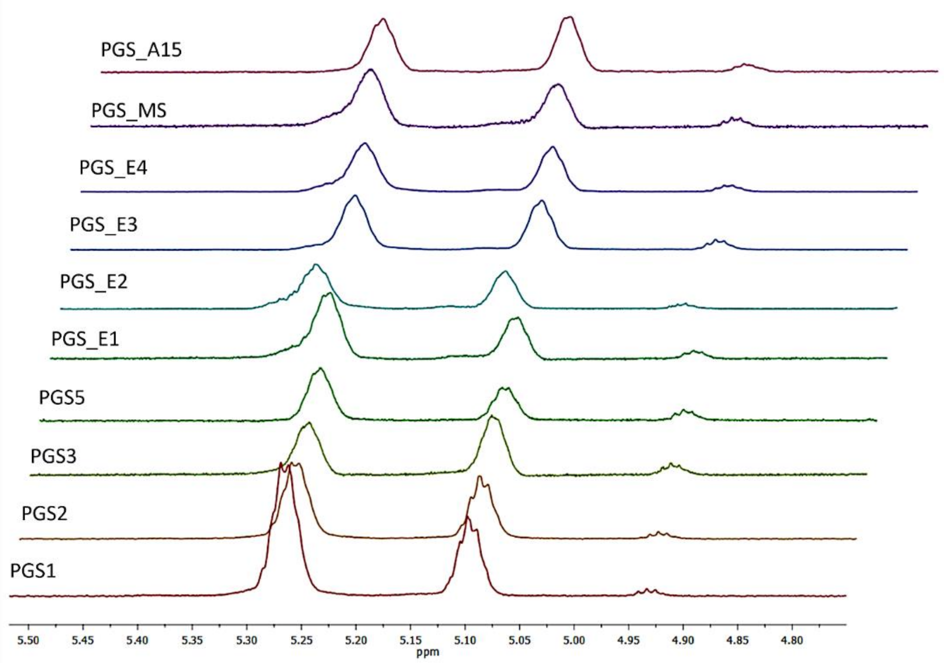

Figure S4. ^{1}H NMR spectra of PGS prepolymers in $CDCl_3$ obtained through different synthetic methods, enlarged region of the free of overlapping signals of the methine protons of the 2G, 1,2G and 1,2,3G units.
